# Audio-Visual Distraction- A Non-Pharmacological Approach to Alleviate Pain in Pediatric Vaccine Administration: An Observational Study

**DOI:** 10.31729/jnma.8856

**Published:** 2025-01-31

**Authors:** Harsshika Bajaj, Akaash Tandel, Uday Rajput, Naresh Sonkawade, Rahul Dawre, Sameer Pawar, Sangeeta Chivale, Pragathi Kamath, Kanchan Sakharkar, Poonam Sancheti, Murlidhar Tambe, Aarti Kinikar

**Affiliations:** 1 Byramjee Jeejeebhoy Government Medical College, Pune, Maharashtra, India; 2 Department of Pediatrics, Byramjee Jeejeebhoy Government Medical College, Pune, Maharashtra, India; 3 Department of Community Medicine, Byramjee Jeejeebhoy Government Medical College, Pune, Maharashtra, India

**Keywords:** *audio-visual*, *children*, *FLACC*, *pain*, *vaccination*

## Abstract

**Introduction:**

The routine vaccine injections are one of the most common and painful procedures during childhood specifically in infancy. In order to improve the effectivity of pain reduction during routine vaccination OPD this study was done to analyse the effectiveness of audio-visual gadgets as a distraction tool for pain reduction in infants during the vaccination procedure.

**Methods:**

A comparative observational study conducted at a tertiary healthcare center vaccination out patient department. Children aged 1 month to 2 years receiving the vaccine were included in the study. The study group was exposed to audio-visual clip while the control group received the vaccine as per routine vaccination protocol. The Face, Legs, Activity, Cry, Consolability Scale score was used to assess the behavioural reactions to pain which assesses five behavioural areas. The study protocol was approved by the Institute Ethics Committee (Reference number: BJGMC/IEC/1122238-238).

**Results:**

The Face, Legs, Activity, Cry, Controllability Scale score were studied among two groups, it was observed that the pain score and every component of the score had a statistically significant lower mean score in audio-visual group as compared to the control group. Mean pain score in the audio-visual group was 6.31±0.79 as compared to 9.57±0.65 in the control group with the p value of 0.001.

**Conclusions:**

A lower pain score in response to vaccination with an audio-visual distraction indicates that it can be regularly used to reduce pain during the vaccination procedure and can be used during different painful procedures.

## INTRODUCTION

Vaccination is a crucial aspect of Pediatric healthcare during childhood. Multiple injections induce iatrogenic pain, leading to alterations in pain perception, anxiety, fear, and avoidance behaviours toward medical interventions in future.^1-4^ Many pharmacological and non-pharmacological strategies are explored to attenuate vaccination-related pain. Among these, distraction emerges as a promising method, various forms of distraction techniques include watching cartoons, utilizing party blowers etc.^5-10^ Some studies advocate the use of skin-to-skin contact and breastfeeding to mitigate pain in infants, but contradictory findings have also been reported.^9-12^

Despite the diverse range of distractions, variability in their effectiveness remains, due to subjective selfreporting measures.^13^ The utilization of objective assessment tools, like the Face, Legs, Activity, Cry, Consolability (FLACC) Pain Scale, offers a more standardized approach to it.^14^ So, our study aims to investigate the effectiveness of audio-visual distraction in reducing pain during routine childhood vaccination without altering established vaccination protocols.

## METHODS

This comparative observational study was conducted at a tertiary healthcare centre situated in western Maharashtra, India from December 2022 to March 2023. The study protocol was approved by the Institute Ethics Committee (Reference number: BJGMC/IEC/1122238-238). Children aged between 1 month to 2 years visiting the vaccination outpatient department (OPD) and receiving intramuscular injections were eligible for inclusion. Exclusion criteria comprised children with neurological or chronic disorders, malnourished children, those who had received analgesic medication within three hours before vaccination, and those who had not been fed within one hour before vaccination. A sample size of 44 children in both the control and study groups was calculated using power analysis. Considering a type I error rate of 0.05 and 80% power, a sample size of 37 per group was deemed necessary to detect a 1.6-point reduction in the FLACC score with a standard deviation of 2.4, based on findings from a previous study. ^15^

Those parents who volunteered to participate were explained the study and written informed consent was obtained before collecting the data. The study procedure was conducted without interrupting the standard vaccination protocol of the centre. The participant record form and FLACC Pain Scale were used in data collection. This form comprised participant's demographic data, such as, gender, age, and weight. Children were allocated to the study group if they attended the OPD on Monday, Wednesday, and Friday, while those attending on Tuesday, Thursday, and Saturday constituted the control group. The nurse was instructed to follow the same steps and attitude towards children in both groups, to avoid bias. The preparation and actual procedure were consistent for all the infants. All infants were awake and had clean diapers at the time of injection. During the vaccination procedure, parents in both groups were not permitted to calm their infants through touch or verbal reassurance. The injection site was cleaned with alcohol and allowed to air dry before the needle was inserted. The vaccination was administered, an intramuscular injection with a 24-gauge needle into the vastus lateralis muscle, at a 90° angle. The duration of the intramuscular injection was approximately 10 seconds (for the standard technique, the needle was inserted at 90 degrees with steady pressure and aspiration was performed. The vaccine was slowly injected over 5-10 seconds and the needle was then slowly withdrawn). A light pressure was applied to the site after injection.

An audio-visual (AV) clip of an English nursery rhyme was shown to children in the study group. The AV clip was displayed on an Android tablet with a screen size of 15*7 cm and a sound intensity of 80 decibels kept at 1 feet distance from the child. The duration of exposure to the AV clip was at least 30 seconds before vaccination. One investigator held the vaccinated leg of the child while another assessed the child's response using the FLACC pain scale. The FLACC was used to assess the behavioral reactions to pain by infants and children. The FLACC pain scale was assessed by five behavioral areas (facial expression of the child, the position of the legs, activity, crying, and consolability) with scores ranging from 0 to 2 for each item. Using the FLACC Pain Scale, the child's behavioural reactions to pain during the procedure were determined in the control and study groups. These responses were recorded by the researcher.

Data analysis included chi-square tests for group homogeneity, t-tests for intra- and intergroup FLACC score differences and mean and percentage distributions for infant characteristics determining the intergroup differences in the individual characteristics of the FLACC parameters in the study and control groups. Data analysis was done using IBM SPSS Statistics for Windows, version 21 (IBM Corp., Armonk, N.Y., USA) Statistical significance was considered at a P-value less than 0.05.

## RESULTS

In the present study, 90 children were enrolled for study purposes and were divided into control and intervention (AV Gadget) group equally. Out of these, 45 control group participants 22 (48.89%) were male and 23 (51.11%) were female similarly among intervention group 25 (55.56%) were male and 20 (44.44%) were female. There was no significant difference in gender distribution among study groups (p>0.05). Similarly, on comparison of weight and age category, there was no significant difference among study groups (p>0.05), (Table 1).

**Table 1:** Demographic characteristics of Study & Control group (n=90).

Variables	AV gadgets (n=45) n(%)	Control (n=45) n(%)	P valve
Male (n= 47)	25 (55.55)	22 (48.88%)	0.673
Female (n= 43)	20 (44.44)	23 (51.11%)	
Weight	<5kg (n= 40)	22 (48.88)	18 (40.00%)	0.525
	>5kg (n = 50)	23 (51.11)	27 (60.00%)	
Age	<6 months (n= 64)	33 (73.33)	31 (68.88%)	0.816
	>6 months (n= 26)	12 (26.66)	14 (31.11%)	
Mean age (Months) (n= 90)		5.74	5.32	0.697
Mean weight (kg ± SD) (n= 90)		5.82±1.61	5.87±1.54	0.895

AV = audio-visual, kg = Deviation Kilogram, SD = Standard Deviation

The FLACC score comparison between study group vs control group showed that mean score in relation to gender, weight, and age it was less in study group as compared to control group. On applying t test of significance there was statistically high significance observed in mean distribution of FLACC score among study groups (p<0.05), (Table 2).

**Table 2:** Comparisons of the mean FLACC pain scores among study group v/s Control group (n=90).

Variables		AV Gadgets- FLACC score (Mean score± SD)	Control- FLACC score (Mean score± SD)	P value
Male		6.4 ±0.95	9.64±0.65	0.001
Female		6.25±0.78	9.52±0.66	0.001
Weight	<5kg	6.45±0.58	9.5±0.70	0.001
	>5kg	6.22±0.90	9.63±0.62	0.001
Age	<6 months	6.39±0.64	9.58±0.67	0.001
	>6months	6.17±0.93	9.57±0.64	0.001

Individual components of FLACC score were analysed among two groups, and every component of FLACC score had statistically significant lower mean score in study group as compared to control group. This observation was consistent with the total FLACC score in these two groups (Table 3).

**Table 3:** Intergroup comparisons of the individual parameters of the FLACC score (n=90).

Variables	AV Gadgets (mean score±SD)	Control (mean score±SD)	Test and significance (P value)
Face score	1.42±0.50	1.98±0.15	0.0001
Legs score	1.44±0.50	1.89±0.32	0.0001
Activity score	1.22±0.42	1.76±0.44	0.0001
Cry score	1.27±0.45	1.98±0.15	0.0001
Consolability score	0.96±0.21	1.96±0.21	0.0001
Total FLACC score	6.33±0.88	9.58±0.66	0.0001

## DISCUSSION

Based on the results of this observational study, it is found that audio-visual gadget proves to be an effective mode of distraction and hence helps to significantly reduce the pain score as measured by the FLACC score. This study has selected age group of 1 month to 2 years considering more appropriate way of assessing and analysing FLACC scores with physiological development of audio-visual coordination in this age groups as compared to studies done by Funda K. et al 2012 which included children above 38 weeks and Park et al in 2022, which included children between 6 months to 4 years.^14^

Park et al, 2022 showed VR using a domed ceiling screen could be an effective distraction method only for girls, especially in the preprocedural stage, while our study showed a significant decrease in the pain score in both genders. ^15^

Previous studies addressing nonpharmacological methods, such as parental holding, sucrose, and breastfeeding confirmed pain reduction in infants when they were subjected to painful procedures. ^2,5^ However, studies on the effectiveness of distraction to reduce anxiety and pain in infants are sparse and have mixed results.^11^ In this study, the efficacy of distracting infants and children by using audio-visual device during vaccination revealed a significant reduction in pain. Assessment of the individual parameters of the FLACC score also showed that the use of AV gadgets has a significant impact on reducing the individual pain parameters as well. Pain reduction was also reported in previous studies with various distraction methods during vaccination.^6,7,8^ The device used in the present study is clinically significant, because it is multi-sensorial and moreover very practical to use.^14,15,16^ It has been reported that distraction strategies which use two senses (visual with audio) appear to be more effective at reducing pain as compared to the use of either one alone; the content, intensity, and combinations of multisensory stimuli are important elements of distraction interventions.

Despite achieving less pain with distraction via an AV gadget, the current study had some limitations as pain score being subjective and based on observational measures. Physiological indices (e.g., heart rate, blood pressure, and oxygen saturation) could have been assessed to more accurately determine this factor. Bias of the child to an audio visual can also pose to be a confounding factor, especially in children above 1 year of age.

## CONCLUSIONS

In conclusion, some audio-visual gadgets with both visual and auditory elements can be used as a measure of distracting infants from experiencing pain during vaccination.
